# Lung ultrasound score assessing the pulmonary edema in pediatric acute respiratory distress syndrome received continuous hemofiltration therapy: a prospective observational study

**DOI:** 10.1186/s12890-021-01394-w

**Published:** 2021-01-25

**Authors:** Fei Wang, Chunxia Wang, Jingyi Shi, Yijun Shan, Huijie Miao, Ting Sun, Yiping Zhou, Yucai Zhang

**Affiliations:** 1 Department of Critical Care Medicine, Shanghai Children’s Hospital, Shanghai Jiao Tong University, Shanghai, 200062 China; 2 Institute of Pediatric Critical Care, Shanghai Jiao Tong University, No.355 Luding Road, Putuo District, Shanghai, 200062 China

**Keywords:** Lung ultrasound score, Acute respiratory distress syndrome, Continuous renal replacement therapy, Child

## Abstract

**Background:**

Lung ultrasound score is a potential method for determining pulmonary edema in acute respiratory distress syndrome (ARDS). Continuous renal replacement therapy (CRRT) has become the preferred modality to manage fluid overload during ARDS. The aim of this study was to evaluate the value of lung ultrasound (LUS) score on assessing the effects of CRRT on pulmonary edema and pulmonary function in pediatric ARDS.

**Methods:**

We conducted a prospective cohort study in 70 children with moderate to severe ARDS in a tertiary university pediatric intensive care unit from January 2016 to December 2019. 37 patients received CRRT (CRRT group) and 33 patients treated by conventional therapy (Non-CRRT group). LUS score was measured within 2 h identified ARDS as the value of 1st, and the following three days as the 2nd, 3rd, and 4th. We used *Spearman* correlation analysis to develop the relationship between LUS score and parameters related to respiratory dynamics, clinical outcomes as well as daily fluid balance during the first four days after ARDS diagnosed.

**Results:**

The 1st LUS score in CRRT group were significantly higher than Non-CRRT group (*P* < 0.001), but the LUS score decreased gradually following CRRT (*P* < 0.001). LUS score was significantly correlated with Cdyn (dynamic lung compliance) (1st: *r* = − 0.757, 2nd: *r* = − 0.906, 3rd: *r* = − 0.885, 4th: *r* = − 0.834), OI (oxygenation index) (1st: *r* = 0.678, 2nd: *r* = 0.689, 3rd: *r* = 0.486, 4th: *r* = 0.324) based on 1st to 4th values (all *P* < 0.05). Only values of the 3rd and 4th LUS score after ARDS diagnosed were correlated with duration of mechanical ventilation [1st: *r* = 0.167, *P* = 0.325; 2nd: *r* = 0.299, *P* = 0.072; 3rd: *r* = 0.579, *P* < 0.001; 4th: *r* = 0.483, *P* = 0.002]. LUS score decreased from 22 (18–25) to 15 (13–18) and OI decreased from 15.92 (14.07–17.73) to 9.49 (8.70–10.58) after CRRT for four days (both *P* < 0.001).

**Conclusions:**

LUS score is significantly correlated with lung function parameters in pediatric ARDS. The improvement of pulmonary edema in patient with ARDS received CRRT can be assessed by the LUS score.

*Trial registration* CCTR, ChiCTR-ONC-16009698. Registered 1 November 2016, prospectively registered, http://www.chictr.org.cn/edit.aspx?pid=16535&htm=4. This study adheres to CONSORT guidelines.

## Background

Non-cardiogenic pulmonary edema is one of the main forms of presentation caused by non-cardiogenic factors such as shock, sepsis, pneumonia, and other in ARDS [1]. Reduction of pulmonary edema is critical for improving pulmonary function, and assessment of pulmonary edema is effective method in monitoring and guidance of therapy in patients with ARDS.

Though lung computed tomography (CT) is the gold standard for noninvasive evaluation of pulmonary edema, it is unsuitable to perform repeatedly in children with severe ARDS due to its radioactive hazard and safety [2]. Extravascular lung water (EVLW) which results in pulmonary edema reflects the severity of ARDS [3]. But detection of EVLW by a pulse indicator continuous cardiac output (PiCCO_2_) device is invasive and inconvenient. Moreover, evidences demonstrated that the relationship between EVLW and PaO_2_/FiO_2_ ratio or oxygenation index (OI) [4] was weak. Otherwise, LUS could determine nearly all of pulmonary pathologic abnormalities. Recent reports indicated that LUS score is used as an alternative method for evaluating pulmonary edema and EVLW in ARDS [5–7]. However, there is no report about the relationship between LUS score and pulmonary function in children with ARDS. Our previous study and other report indicated that patients with ARDS received CRRT had better outcome than that without CRRT [8, 9].

We hypothesized that LUS score could assess accurately lung function and improvement of pulmonary edema during CRRT in pediatric ARDS.

## Methods

### Patients

A prospective cohort study was conducted, and children with moderate to severe ARDS admitted to pediatric intensive care unit (PICU) were enrolled from January 2016 to December 2019 at Shanghai Children's Hospital. Moderate to severe ARDS was defined according to the PALICC definition of pediatric ARDS [4]. The exclusion criteria included: (1) patients who were in PICU less than 72 h; (2) patients with lack of appropriate acoustic window; (3) patients with pneumothorax; (4) patients with hypoxemia secondary to cardiac disease congenital cardiovascular disease or chronic cardiopulmonary disease. According to whether CRRT was used during PICU stay, patients were divided into CRRT group and Non-CRRT group.

All the patients were received the mechanical ventilation based on lung protective-ventilation strategy or/and prone positioning, neuromuscular blockade (NMB), conservative fluid management [10]. The total fluid volume was generally 60–70 ml/kg d or 1200–1500 ml/m^2^ d, blood transfusion if hemoglobin level down to 7.0 g/dL during PICU stay [11]. Patients received diuretics according to daily fluid balance when patients didn’t receive CRRT.

The study protocol was approved by the local ethics committee of Children's Hospital affiliated to Shanghai Jiao Tong university (Approval number: 2016R007-E03). The informed consent was signed by the patients’ parents or relatives.

### Lung ultrasound score

LUS was performed using a 13–6 MHz transducer (M-Turbo Ultrasound System, Mini-Dock-M Series, SonoSite). According to previous studies [8, 9, 12, 13], patients were examined in supine, lateral, and prone positions applying the probe perpendicularly to the chest wall surface in order to get the longitudinal scan. Each hemithorax is divided into three regions by sternum, anterior and posterior axillary lines, and each region is divided into upper and lower halves. Each region should be correctly identified the pleura lines and A line by the linear probe in longitudinal scan.

Twelve areas are identified in turn and each region is assigned scores from 0 to 3. The LUS score is the sum of twelve areas, and the final LUS ranges from 0 to 36. In the present study, the definition of LUS score [14] and the representative images for different scores were shown (Table 1). The pathophysiological changes were described with different ultrasonic signs [15]. All of the images and clips were collected and evaluated by two PICU expert physicians independently who had been trained and could complete lung ultrasound skillfully. All of the images, physical characteristics, baseline data and treatment of patients were all anonymous when they were evaluated by these operators.

**Table 1:**
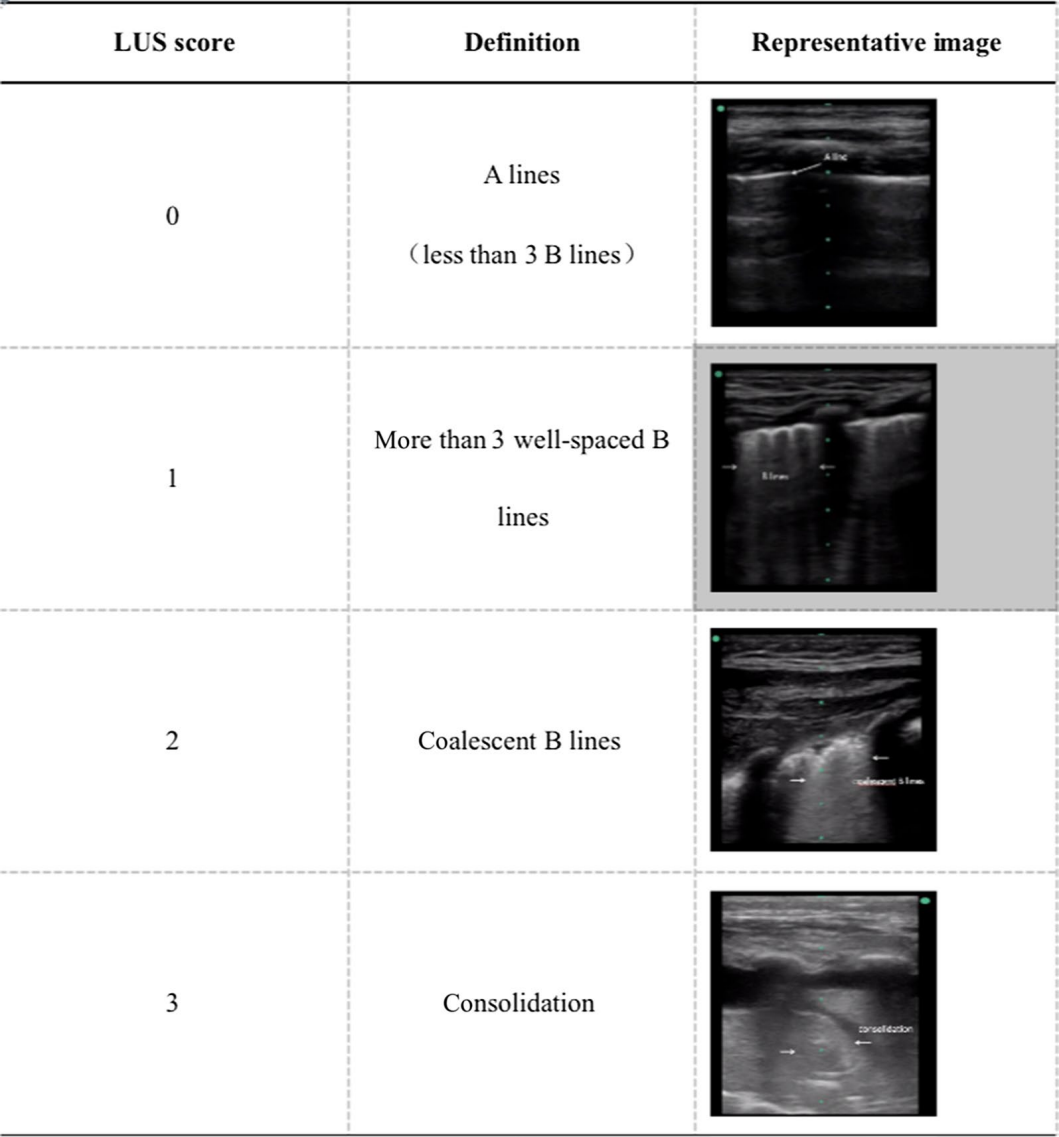
Definition of LUS score and representative images in this study

### CRRT and mechanical ventilation

The CRRT mode was continuous veno-venous hemofiltration (CVVH) using Prismed or Prismaflex M60/100 membrane hemofilter equipped with an AN69 (Gambro Renal Products, Meyzieu, France) in a multifiltrate continuous renal replacement therapy machine (Gambro or Gambro prismaflex, Gambro Lundia Monitor Division, Lund, Sweden). The indications for CRRT initiation include sepsis complicated by moderate or severe ARDS (PaO_2_/FiO_2_ < 150 mmHg), AKI, or fluid overload (> 10%). The performance and management for CRRT were described as our previous study [16].

The modality of mechanical ventilation was intermittent mandatory ventilation (IMV) with PEEP levels 8–15cmH_2_O and positive inspiration pressure (PIP) based on target tidal volume (Vt) of 4–8 ml/kg [11]. Parameters were aligned with lung protective ventilation strategy when patients met the diagnosis of moderate to severe pediatric ARDS.

### Data collection

**Fig. 1:**
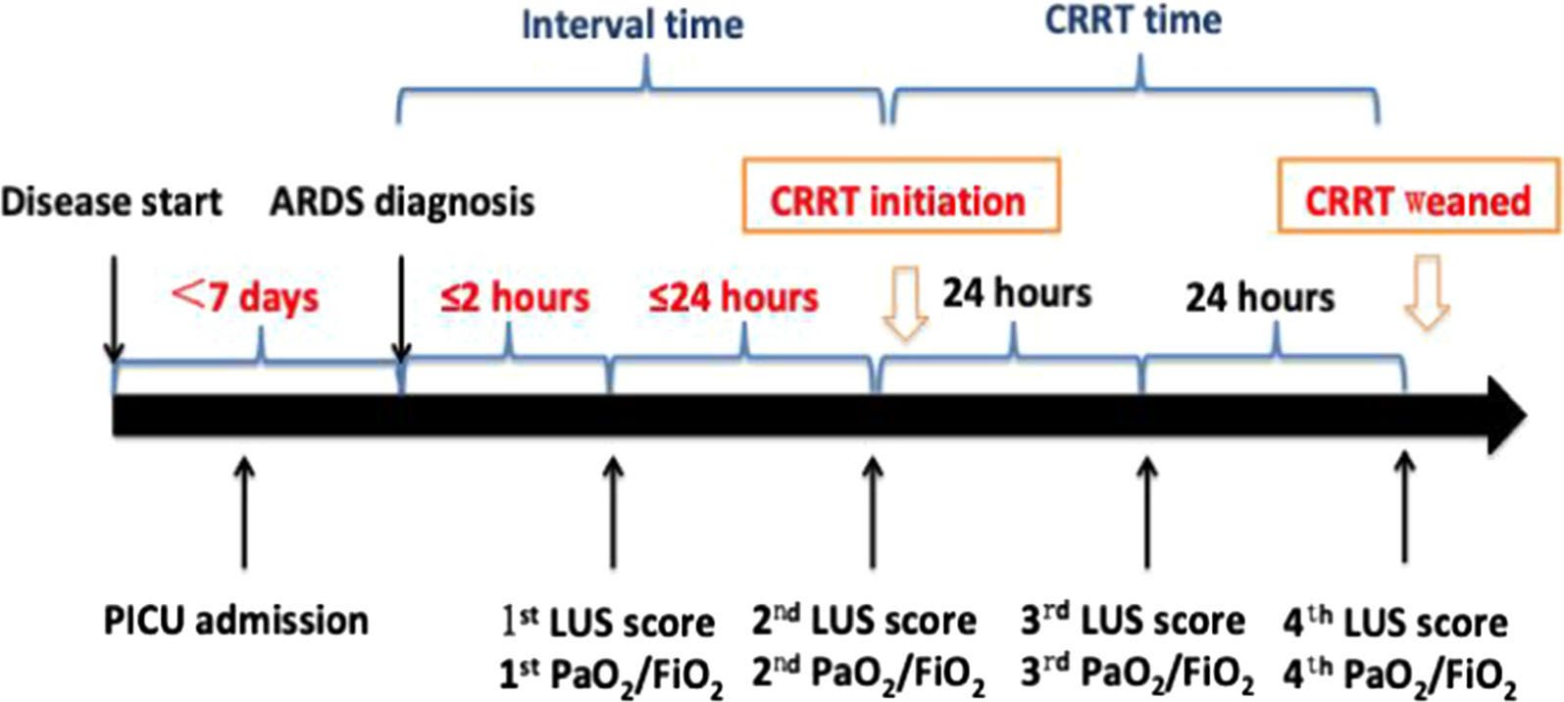
The schematic diagram of LUS score determination

Demographic data such as age, sex, and body mass index (BMI), the pediatric risk mortality III (PRISM III) score [17] and co-morbidity were collected on PICU admission. Clinical parameters including fractional concentration of oxygen in inspired gas (FiO_2_), PaO_2_/FiO_2_, PaCO_2_, OI, dynamic lung compliance (Cdyn) which was continuously displayed using ventilators (MAQUET company, Servo-i serious) [18, 19]. MV settings including PIP, PEEP, and FiO_2_ were collected while measuring LUS score from identified ARDS to the following three days. Daily fluid balance information and hospital mortality were collected. LUS score was determined within 2 h after moderate to severe ARDS diagnosed as the value of 1st, then measured every morning in following three days as the values of 2nd, 3rd, and 4th. The schematic diagram of LUS score determination was shown in Fig. 1. In addition, duration of mechanical ventilation, duration of CRRT, length of PICU or hospital stay were recorded.

### Statistical analysis

The data were performed with SPSS 17.0 statistics (SPSS Inc, Chicago). The characteristics of the patients were reported as percentages for categorical variables and compared the differences between groups by *chi*-square test. The continuous data with abnormal distribution were expressed as median (interquartile range, IQR) and compared using the Mann–Whitney *U* test. The correlation between LUS score and mechanical ventilation (MV) duration, length of PICU stay, Cdyn, PaCO_2_, OI and the correlation between the change in LUS scores and the change in daily fluid balance volume during the four days after ARDS diagnosed were all performed using *Spearman* correlation analysis. Friedman test was used to compare mean of more than 2 sets of data. *P* value < 0.05 was considered to be statistically significant.

## Results

### Baseline characteristics of patients

**Fig. 2:**
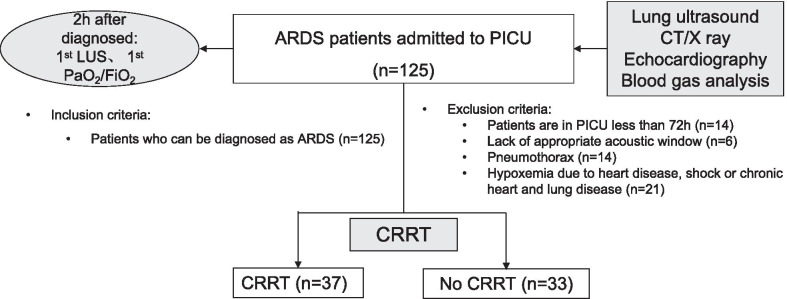
Flowchart for patients’ enrollment

A total of 125 children with moderate to severe ARDS admitted to PICU were eligible from January 2016 to December 2019. Among them, there were 14 cases with less than 72 h in PICU, and 6 patients were lack of appropriate acoustic window, 14 cases were pneumothorax and 21 patients were hypoxemia secondary to cardiac disease congenital cardiovascular disease or chronic cardiopulmonary disease. Finally, 70 children were enrolled in this study (Fig. 2). The median age was 33 (10–52) months and 41 cases were male (58.57%). The hospital mortality rate was 38.57% (27/70). There were 37 patients in the CRRT group, and 33 patients in the None-CRRT group. The baseline characteristics and outcome of patients were summarized in Table 2. The PRISM III score, mechanical ventilation settings (PIP, PEEP and FiO_2_), proportion of complication with AKI, OI, mechanical ventilation duration, length of PICU stay in the CRRT group were significantly higher than that in the Non-CRRT group (*P* < 0.05, Table 2). There were no significant differences in age, gender, BMI, PaCO_2_ and hospital mortality (all *P* > 0.05, Table 2).

**Table 2:** Baseline characteristics and outcome of patients with moderate to severe acute respiratory distress syndrome in this study

Characteristics	Non-CRRT (n = 33)	CRRT (n = 37)	*P* value
Age, month	24 (9–53)	35 (12–52)	0.667
Male, n (%)	19 (57.58)	22 (59.46)	0.873
PRISM III	15 (12–18)	17 (15–20)	0.016
BMI, kg/m (2)	14.70 (12.65–16.86)	14.00 (12.70–16.38)	0.778
Co-morbidity, n (%)			
Immune system disease	1 (3.03)	4 (10.81)	0.207
Genetic metabolic disease	1 (3.03)	0 (0)	0.286
Leukemia/tumor	6 (18.18)	7 (18.92)	0.937
Complication, n (%)			
Hepatic failure	2 (6.06)	1 (2.70)	0.489
AKI	9 (27.27)	25 (67.57)	0.001
Brain dysfunction	1 (3.03)	1 (2.70)	0.935
Gastrointestinal dysfunction	2 (6.06)	3 (8.11)	0.740
Pancreatitis	0 (0)	2 (5.41)	0.175
OI	7.69 (6.46–9.50)	13.15 (10.00–14.78)	< 0.001
PaCO_2_, mmHg	55.00 (38.00–61.00)	45.00 (34.50–62.00)	0.225
Mechanical ventilation settings			
PIP, cmH_2_O	26 (25–28)	29 (27–31)	< 0.001
PEEP, cmH_2_O	9 (8–10)	12 (11–14)	< 0.001
FiO_2_	0.55 (0.50–0.74)	0.70 (0.60–0.88)	0.002
Duration of mechanical ventilation, day	6 (4–9)	8 (6–15)	0.012
Length of PICU stay, day	10 (6–17)	14 (10–20)	0.047
Length of hospital stay, day	19 (12–30)	24 (17–35)	0.081
Hospital mortality, n (%)	11 (33.33)	16 (43.24)	0.395

### Correlation of LUS score to OI, PaCO_2_, dynamic lung compliance and fluid balance

**Fig. 3:**
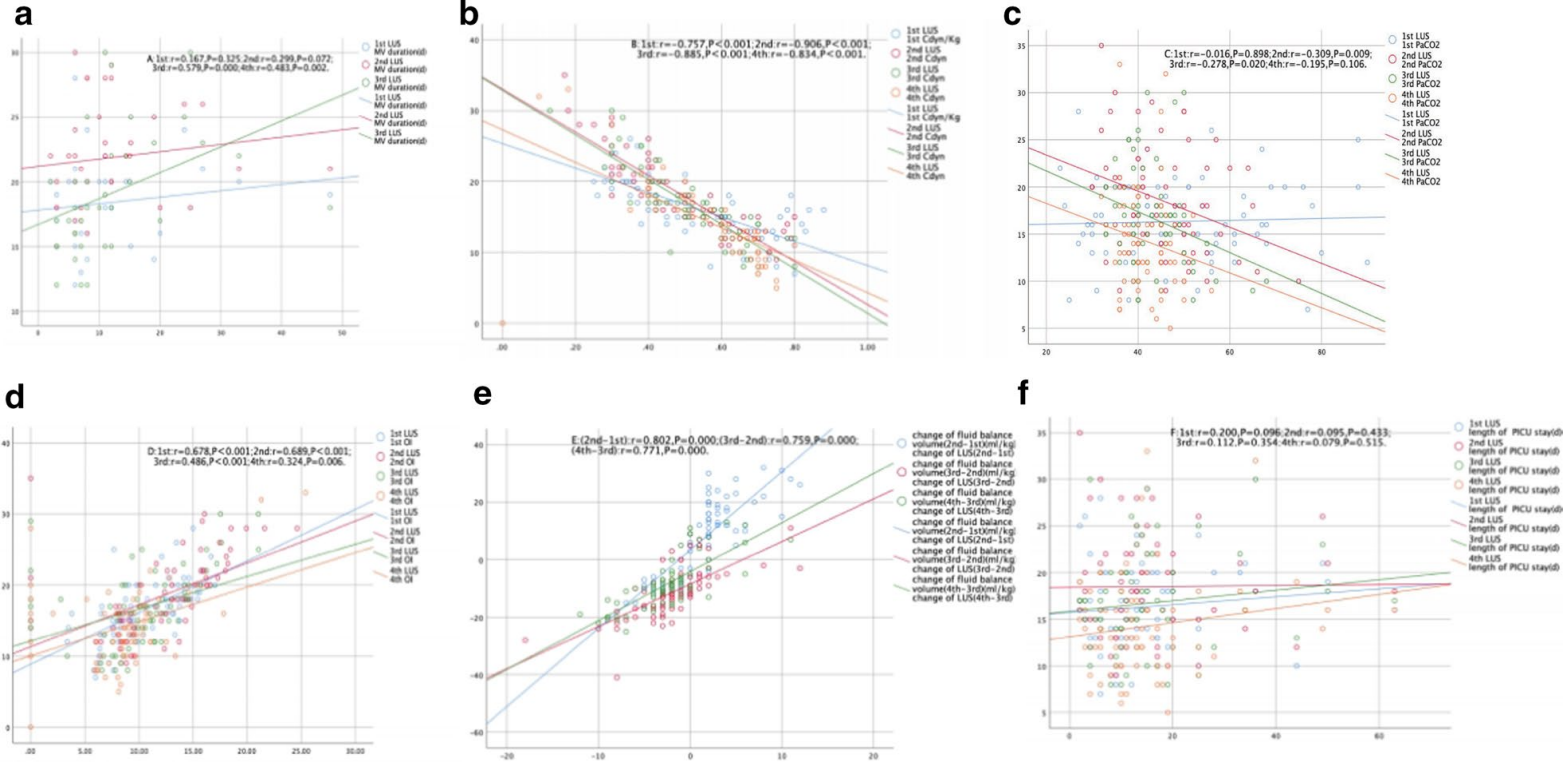
Scatterplots demonstrating the correlation between LUS score and parameters related to respiratory dynamics or clinical outcomes in patients with ARDS. **a** MV duration, **b** Cdyn, **c** PaCO_2_, **d** OI, **e** change of fluid balance volume, **f** length of PICU stay

Except for patients who were forced to wean from mechanical ventilation because of death, only LUS score based on 3rd and 4th values were positively correlated with duration of mechanical ventilation [1st: *r* = 0.167, *P* = 0.325, 2nd: *r* = 0.299, *P* = 0.072, 3rd: *r* = 0.579, *P* < 0.001, 4th: *r* = 0.483, *P* = 0.002] (Fig. 3a). LUS score was negatively correlated with Cdyn [1st: *r* = − 0.757, 2nd: *r* = − 0.906, 3rd: *r* = − 0.885, 4th: *r* = − 0.834, all *P* < 0.001] or PaCO_2_ [1st: *r* = − 0.016, *P* = 0.898; 2nd: *r* = − 0.309, *P* = 0.009; 3rd: *r* = − 0.278, *P* = 0.02; 4th: *r* = − 0.195, *P* = 0.106] based on 1st to 4th values after ARDS diagnosed (Fig. 3b, c).

Positive relationships were observed between LUS score and OI [1st: *r* = 0.678, 2nd: *r* = 0.689, 3rd: *r* = 0.486, 4th: *r* = 0.324, all *P* < 0.05] during the first four days after ARDS diagnosed (Fig. 3d). The change in daily fluid balance volume was positively correlated with the change in LUS score during four days [(2nd–1st): *r* = 0.802, (3rd–2nd): *r* = 0.759, (4th–3rd): *r* = 0.771, all *P* < 0.001] (Fig. 3e). However, there were no correlations between LUS score and length of PICU stay (Fig. 3f).

The median of LUS score, OI, PEEP, Cdyn in the CRRT group were significantly different during the first four days after identified ARDS (all *P* < 0.001, Table 3). CRRT group displayed peak median value of LUS score and OI one day after diagnosis as ARDS. Thirty-six patients received CRRT on the second day after diagnosis (97.30%). In the Non-CRRT group, there were only significant difference in PaCO_2_ during the first four days (*P* < 0.001, Table 3).

**Table 3:** LUS score, respiratory dynamics, daily fluid balance volume and the change in LUS score in with (CRRT) and without hemofiltration (Non-CRRT) group

Parameters	CRRT (n = 37)					Non-CRRT (n = 33)				
	1	2	3	4	*P*	1	2	3	4	*P*
LUS	18 (16–20)^a^	22 (18–25)^a^	18 (16–22)^a^	14 (12–18)^b^	< 0.001	14 (12–16)	14 (12–16)	14 (10–17)	12 (9–16)	0.154
OI	13.15 (10.00–14.78)^a^	15.85 (13.54–17.46)^a^	11.63 (10.24–14.47)^a^	9.47 (8.63–10.50)^b^	< 0.001	7.69 (6.46–9.50)	8.84 (8.25–10.18)	8.60 (7.56–11.05)	8.12 (6.85–10.45)	0.242
PaCO_2_, mmHg	45 (35–62)	42 (38–50)	40 (38–45)^b^	40 (38–45)	0.078	55 (38–61)	46 (41–52)	45 (40–50)	42 (38–45)	0.006
Cdyn, ml/cmH_2_O/kg	0.38 (0.31–0.58)^a^	0.40 (0.30–0.42)^a^	0.44 (0.38–0.56)^a^	0.60 (0.51–0.66)	< 0.001	0.61 (0.46–0.72)	0.61 (0.50–0.71)	0.60 (0.44–0.68)	0.63 (0.45–0.70)	0.315
PEEP, cmH_2_O	12 (11–14)^a^	14 (13–15)^a^	12 (11–14)^a^	10 (9–11)^a^	< 0.001	9 (8–10)	9 (8–10)	9 (8–11)	8 (8–10)	0.055
Daily fluid balance volume, ml/kg		17.00 (8.50–22.50)^a^	− 16.00 [− 20.00–(− 12.00)]^a^	− 10.00 [− 16.50–(− 8.50)]^b^	< 0.001		0 (− 9.00–10.50)	− 6.00 (− 10.00–0)	− 7.00 [− 11–5.50]	0.076
Change in LUS score		2 (2–4)^b^	− 3 [− 5–(− 1)]^b^	− 3 [− 6–(− 2)]^b^	< 0.001		1 (− 2–3)	0 (− 3–1)	− 1 (− 3–1)	0.221

The values of daily fluid volume and change in LUS score are as following 2nd–1st, 3rd–2nd, 4th–3rd

^a^*P* < 0.001 for CRRT group vs. Non-CRRT group

^b^*P* < 0.05 for CRRT group vs. Non-CRRT group

**Fig. 4:**
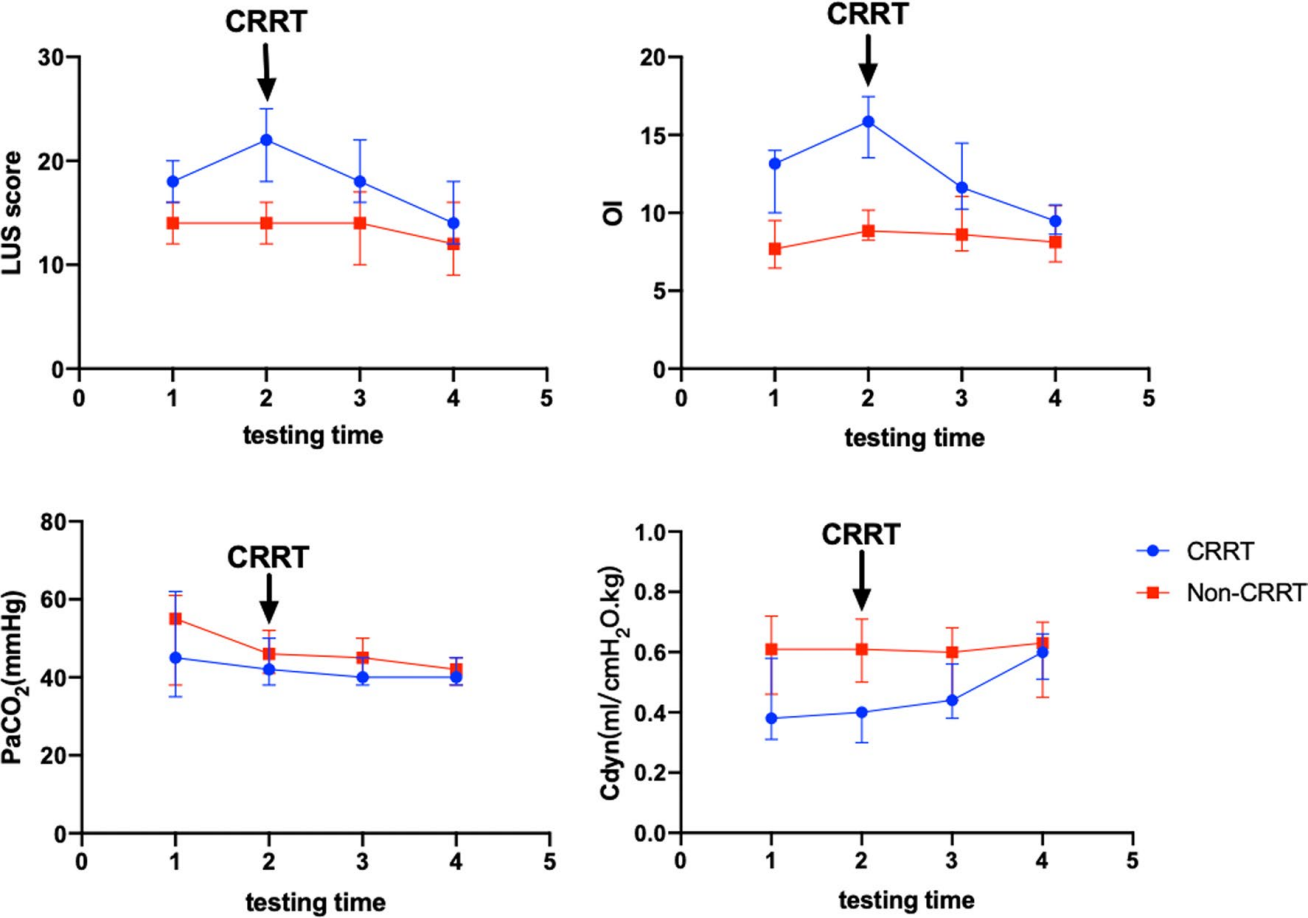
Comparison of LUS score, respiratory dynamics in with (CRRT) and without hemofiltration (Non-CRRT) group. a LUS score, b OI, c PaCO_2_, d Cdyn

**Fig. 5:**
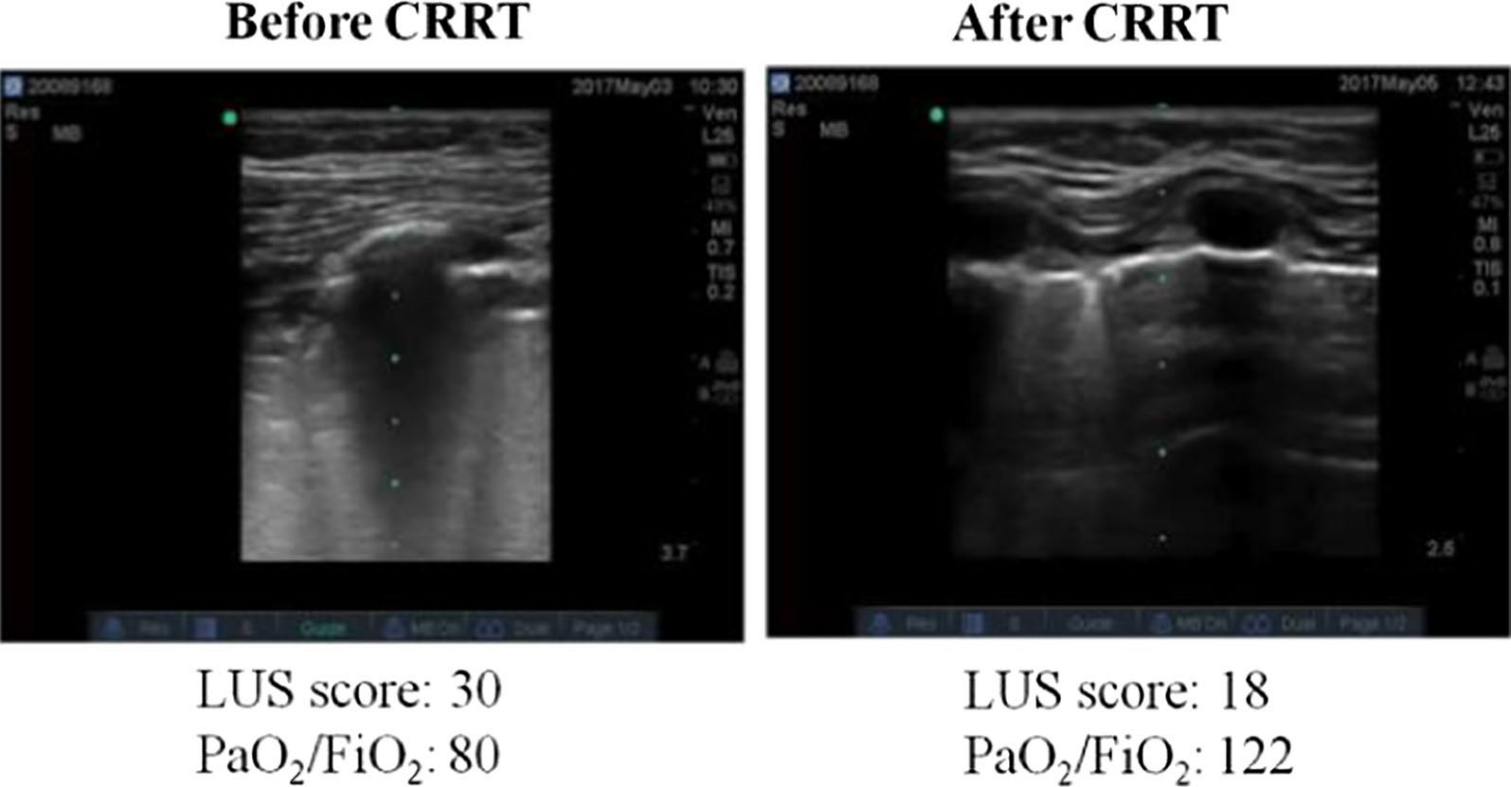
The representative images of LUS before and after hemofiltration

Though the median of LUS score and OI in the CRRT group were higher than the Non-CRRT group during the first four days, but on the 4th day the difference between two groups decreased. The paired analysis revealed that Cdyn in the CRRT group were significantly lower than that in the Non-CRRT group in first three days after diagnosis as ARDS (*P* < 0.001, Table 3). In the CRRT group, Cdyn increased after received CRRT and compared to Non-CRRT group, there were no significant difference between two groups on the 4th day (Fig. 4). The representative images of LUS were presented as Fig. 5.

The interval time between identified moderate to severe ARDS and CRRT initiation was 6.0 (3.0–10.5) hours, and the median duration of CRRT was 49.5 (45.0–53.5) hours. The median of LUS score [22 (18–25) vs. 15 (13–18)], OI [15.92 (14.07–17.73) vs. 9.49 (8.70–10.58)], and Cdyn [0.40 (0.30–0.42) ml/cmH_2_O/kg vs. 0.60 (0.51–0.65) ml/cmH_2_O/kg] were determined at initiation and after CRRT target weaned in the CRRT group. These results indicated that LUS score, OI and PEEP after CRRT weaned were significantly lower, and Cdyn were increased (*P* < 0.001, Table 4). Otherwise, only the value of PaCO_2_ was decreased on the 4th day after ARDS diagnosis in the Non-CRRT group (*P* = 0.006, Table 4).

**Table 4:** LUS score, respiratory dynamics in with (CRRT) and without hemofiltration (Non-CRRT) group

	CRRT (n = 37)			Non-CRRT (n = 33)		
	Pre-CRRT	Post-CRRT	*P*	1st day	4th day	*P*
LUS	22 (18–25)	15 (13–18)	< 0.001	14 (12–16)	12 (9–16)	0.154
OI	15.92 (14.07–17.73)	9.49 (8.70–10.58)	< 0.001	7.69 (6.46–9.50)	8.12 (6.85–10.45)	0.242
PaCO_2_, mmHg	41 (38–50)	40 (38–45)	0.102	55 (38–61)	42 (38–45)	0.006
Cdyn, ml/cmH_2_O/kg	0.40 (0.30–0.42)	0.60 (0.51–0.65)	< 0.001	0.61 (0.46–0.72)	0.63 (0.45–0.70)	0.315
PEEP, cmH_2_O	14 (13–15)	10 (9–11)	< 0.001	9 (8–10)	8 (8–10)	0.220

## Discussion

In this study, we found that LUS score is correlated with Cdyn, OI in pediatric ARDS, and the changes of LUS score are associated with the improvement of fluid balance and pulmonary function by CRRT intervention or diuretics in pediatric ARDS.

Lung ultrasound is a convenient and repeatable approach used in critically ill patient’s bedside nowadays. In the last decade, a meta-analysis showed that lung ultrasound had a high sensitivity and specificity in critically ill patients who were characterized by pulmonary pathology [20], especially in aspect of assessing EVLW [21, 22]. The association of EVLW determined by PiCCO_2_ with PaO_2_/ FiO_2_ or OI is affected by BW, height, or PEEP [23–26]. According to previous report, LUS as a convenient, noninvasive and portable technique could detect various pathophysiological changes including lung edema and derecruited lung [9, 13, 27], and LUS can detect the increased EVLW by the appearance of B-lines in ARDS [28]. It is worth noting that LUS score is correlated with OI and Cdyn during the first four days after ARDS diagnosis in the present study. The data in our study provides support for the suggestion that LUS score could be a way to quantify the oxygenation state, compliance of lung and the requirement of ventilation pressure in pediatric ARDS. LUS score might more sensitive and specific for assessing pulmonary function compared with invasive determination of EVLW by PiCCO_2_. Moreover, B-lines increased and could be detected despite a normal PaO_2_/FiO_2_ ratio in animal model of lung injury [29], suggesting that LUS score is an early indicator for assessing pulmonary function. Furthermore, the child's thorax is smaller and the chest wall is thinner. So, the visualization of pulmonary ultrasound in longitudinal scan is better than that of adults. To our knowledge, this is the first report about the LUS score as a potential tool for EVLW measurement in estimating pulmonary function in a large pediatric population with ARDS.

It is well known that fluid overload and AKI were the mainly indications for CRRT intervention in patients with ARDS [30]. Our previous multi-center prospective study found that CRRT could significantly decrease hospital mortality rate in pediatric ARDS secondary to sepsis [16]. The pathophysiology of ARDS is inflammatory storm caused by various insults leads to capillary leak and increased EVLW. According to the standard care of patients with moderate to severe ARDS, the use of hemofiltration might be potential tool for negative fluid balance, which is associated with improved lung function [31]. To date, it is lack of effective tool to accurately evaluate the effects of hemofiltration on pulmonary edema and pulmonary function. LUS score has been proposed for semi-quantification of lung aeration [14]. In our study, all the daily median of LUS score after diagnosed as ARDS in the CRRT group were higher than these in the Non-CRRT group and CRRT group had peak median value of LUS score on one day after diagnosis as ARDS. More importantly, a significant reduction in LUS score disappeared following CRRT, and LUS score were positively correlated with the change in daily fluid balance volume in the CRRT group. All these results implied that CRRT improves pulmonary function partially contributed by improvement of pulmonary edema, which might relate to fluid balance by CRRT. As far as we know, this is first report about the closely relationship between LUS score and the effects of CRRT on pulmonary function.

There are several limitations to be considered. First, our study included in a single PICU and baseline characteristics were different between CRRT and Non-CRRT group, which affected the power of the conclusion. Second, due to its limitation of invasiveness, the relationship between the value of EVLW determined by PiCCO_2_ and LUS score was not analyzed. Third, the finding of that LUS score may be provide a threshold value for the initiation of CRRT in pediatric ARDS would need to be validated in more detailed and larger studies. Nevertheless, the results give a new insight into the benefits from monitoring LUS score to assess the severity and the effect of CRRT on pulmonary function in pediatric ARDS.

## Conclusions

LUS score, as an alternative indicator for pulmonary function, is closely correlated to PaO_2_/FiO_2_, OI and Cdyn in pediatric ARDS. The improvement of CRRT on pulmonary function can be assessed by the LUS score. As an easily repeatable, noninvasive, and quantitative tool for detecting pulmonary edema and other pathological signs, the value of LUS score is worth further investigation in a large pediatric population.

## Data Availability

All data generated or analyzed during this study are included in this published article (and its supplementary information files).

## Abbreviations

ARDS: Acute respiratory distress syndrome
ICU: Intensive care unit
CT: Computed tomography
EVLW: Extravascular lung water
OI: Oxygenation index
BW: Body weight
PEEP: Positive end-expiratory pressure
LUS: Lung ultrasound
AKI: Acute kidney injury
RRT: Renal replacement therapy
CRRT: Continuous renal replacement therapy
PICU: Pediatric intensive care unit
NMB: Neuromuscular blockade
CVVH: Continuous veno-venous hemofiltration
IMV: Intermittent mandatory ventilation
PIP: Positive inspiration pressure
Vt: Tidal volume
BMI: Body mass index
PRISM III: Pediatric risk mortality III
FiO_2_: Fractional concentration of oxygen in inspired gas
Cdyn: Dynamic lung compliance
